# Immunomodulatory dynamics of excretory and secretory products on Th9 immune response during *Haemonchus contortus* infection in goat

**DOI:** 10.1371/journal.pntd.0008218

**Published:** 2020-04-03

**Authors:** Muhammad Ali Memon, Muhammad Ali-ul-Husnain Naqvi, Huang Xin, Liang Meng, Muhammad Waqqas Hasan, Muhammad Haseeb, Shakeel Ahmed Lakho, Kalilixiati Aimulajiang, Yongqian Bu, Lixin Xu, Xiaokai Song, Xiangrui Li, Ruofeng Yan

**Affiliations:** MOE Joint International Research Laboratory of Animal Health and Food Safety, College of Veterinary Medicine, Nanjing Agricultural University, Nanjing, Jiangsu, PR China; Uniformed Services University, UNITED STATES

## Abstract

CD4+ T cells play critical roles in mediating adaptive immunity to a variety of pathogens. Recently, new subset of CD4+T named as T helper 9 cells that express the prototypical interleukin-9 (IL-9) cytokine have been recognized in human and mice models during different parasitic infections. *Haemonchus contortus* is a gastrointestinal nematode of small ruminants which cause high mortality in young animals. During infection, Excretory and Secretary Products (ESPs) are released in the host body. No other study has reported yet on immunomodulatory dynamics of *H*. *contortus* ESPs on Th9 immune response *in vitro* or *in vivo*. In this study, immunomodulatory effects of ESPs (5, 10, 20, 40, 80; μg/mL) incubated with goat PBMCs on Th9 cells, IL-9 immune response and TGF-β/Smad signaling regulator were evaluated *in vitro*. Moreover, for *in vivo* study, goats were infected with different doses (P-800, P-2400, and P-8000) of *H*. *contortus* infective larva (L3) and immunomodulatory effects on Th9 cells, IL-9 immune response and TGF-β/Smad signaling regulator were evaluated at 7, 10, 14, 18, 21, 28 Days Post Infection (DPI). Flow cytometry was performed to evaluate the effects on Th9 cells and quantitative real time polymerase chain reaction was performed to evaluate the IL-9 cytokine transcription level. Additionally, fecal egg counting was also performed in parallel to confirm the infection. All goats were dewormed at 29 DPI and all experiments were also performed at 35 DPI, one week post deworming. The finding indicated that 10, 20, 40, 80 μg/mL concentration of ESPs incubated with goat PBMCs showed significant increase in the production of Th9 cells, signature cytokine IL-9 and expression of TGF-β/Smad signaling regulator as compared to control group *in vitro*.All infected groups showed significant increase in production of Th9 cells and IL-9 cytokine and expression of TGF-β/Smad key genes at 18, 21, and 28 DPI as compared to control group. Likewise, at 14 DPI, P-2400 and P-8000 groups showed significant increase in production of Th9 cells, IL-9 cytokine and expression of TGF-β/Smad key genes. While at 10 DPI, production of Th9 cells and IL-9 was significantly increased in P-2400 & P-8000 groups, and at 7 DPI only P-8000 showed significantly increase in IL-9 production. No immunomodulatory effects were observed at 0 and 3 DPI. Additionally, significant gradually up-regulated key genes expression of TGF-β/Smad signaling regulator in all infected groups confirmed the above results. After deworming, production of Th9 cells, associated immune response and expression of signaling regulator in each group were significantly decreased. Based on this study, it is concluded that Th9 immune response was induced during *H*. *contortus* infection in goat by up-regulation of TGF-β/Smad signaling key genes.

## Author summary

*Haemonchus contortus* is one of the most pathogenic nematodes of small ruminants in tropical and sub-tropical areas of the world. This parasite is responsible for anemia, edema, and death in young animal which can lead to billions of economic losses globally. Excretory and secretory products (ESPs) are produced by the parasite to modulate the immune response and to protect both parasite and the host. Th9 cells are a subset of CD4+ T cells producing IL-9 cytokine. Th9 cells were increasingly recognized for being important in immunity to intestinal infection with helminths. In this study, immunomodulatory effects of ESPs on PBMCs derived Th9 cells, IL-9 cytokine and TGF-**β**/Smad signalling were evaluated *in vitro* and *in vivo*. Furthermore, Th9 cells production was significantly enhanced on 7, 10, 14, 18, 21, and 28 Days Post Infection (DPI), while no effect was observed at 0 and 3 DPI *in vivo*. Moreover, the production of IL-9 and TGF-**β**/Smad Pathway key genes increased gradually from 7 to 28 DPI *in vivo*. After deworming, production of Th9 cells was gradually decreased in each group.

## Introduction

The adaptive immune system is regulated by different naïve CD4+ T-produced T helper (Th) cells after stimulation of different antigens that regulate immune response by producing specific cytokines [1,2]. A new subset of CD4+ T cells has been identified named T helper 9 (Th9) cells that produce interleukin (IL)-9 cytokine. IL-9 is a member of the common γ chain cytokine family, which is associated with the Th2 response and exerts broad effects on many cell types, including mast cells, eosinophil, T cells, and epithelial cells [3,4]. IL-9 plays a fundamental role in the control of helminth infection and pathology [5]. IL-9 activated by the Transforming Growth Factor beta (TGF-β) regulator, which involves inducing and maintaining T-regulatory cells, reducing cytotoxic effector immune response, balancing the tolerogenic and immunogenic forces. TGF-β also plays an important role in various physiological states including cancer and other chronic, inflammatory and allergic respiratory diseases [6–9]. Previously, the important role of the TGF-β regulator has been reported in the differentiation of Th9 cells by activating Smad-2, Smad-3, and Smad-4 [10].

In humans, Th9 cells play a protective role in tumors, allergy, autopsy, asthma and auto-immunity diseases at different states [11–14]. Moreover, different roles of Th9 cells have been reported in animal models during intestinal helminth infection [8–10]. In the previous study, the expression of IL-9 in T cells isolated from *Leishmania major*-infected Balb/c mice was reported [15]. Another study also reported transgenic expression of IL-9 in mice infected with *Trichuris muris* and *Trichinella spiralis* [16,17]. Respectively, *Haemonchus contortus* is the most important gastrointestinal nematode parasite which is responsible for brutal health problems and economic losses in the small ruminant industry worldwide [18,19]. It is a blood-sucking parasite that feeds on blood, results in anemia, dehydration, loss of body weight and even death in young animals [20]. The survival in the host reflects the ability of parasites to evade the host immune responses from the early stages of infection [21,22]. During the parasite life stage transformation, different excretory and secretory products (ESPs) are produced by the parasite to modulate the immune response and to protect both parasite and host. ESPs contain various proteins that are capable of modulating the host immune response and related to the pathogenesis of the parasites [23,24]. Peripheral blood mononuclear cells (PBMCs) consist of several populations of immune cells, included lymphocytes (T cells, B cells and NK cells) and monocytes that play important roles in the immune responses. Successful survival of parasites primarily depends on evading the host immune system by penetrating and multiplying within the host’s cells, varying its surface antigens, eliminating protein coat, and modulating the host immune response [16].

Similarly, an increasing amount of experimental evidence indicates an extensive association of host with *H*. *contortus* infection [25]. In our previous proteomic study, the immunomodulatory effects of *H*. *contortus* ESPs (HcESPs) on goat PBMCs were evaluated [21,26]. Moreover, the immunomodulatory effects of HcESPs on PBMCs derived Th9 cells, their immune response (IL-9), and immune regulator (TGF-β) have not yet been studied.

However, this study was planned to evaluate the immunomodulatory effects of HcESPs on the differentiation of Th9 cells, IL-9 cytokine production, TGF-β/Smad pathway key genes expression *in vitro*, and during different stages and intensity of *H*. *contortus* infection in goat.

## Materials and method

### Ethical statement

The treatments of animals in our research were in conformity with the guidelines of the Animal Ethics Committee, Nanjing Agricultural University, China. All animal experiments were abided by the guidelines of the Animal Welfare Council of China. The protocols of our experiments were approved by the Science and Technology Agency of Jiangsu Province (Approval ID: SYXK (SU) 2010–0005).

### Study population

Trail 1: Local crossbreed goats (n = 25), approximately 6–9 months old, were bought and kept under nematode free condition during summer season. All goats were dewormed with anti-parasitic drug Levamisole (8mg/kg body weight) with two weeks interval to keep goats free from naturally acquired helminths infection. Goats were divided randomly into five experimental groups, group-1, control (N), P-800, P-2400, and P-8000 (n = 5 goats/group) to evaluate Th9 mmune response during *H*. *contortus* infection. Goats of group-1 were orally infected with 10,000 infective stage larvae (L_3_) of *H*. *contortus*. Moreover, experimental groups, P-800, P-2400, and P-8000, were orally infected with 800, 2400 and 8000 *H*. *contortus* L_3_, respectively. A microscopic analysis of fecal samples was performed to confirm the infection. Furthermore, control (N) group was kept uninfected. Heparinized peripheral venous blood samples and fecal samples were collected from P-800, P-2400, P-8000, and control groups at 0, 3, 5, 7, 10, 14, 18, 21 and 28 DPI. At 29 DPI, goats of all groups were dewormed and collected the blood again at 35 DPI.

Trial 2: Similar to trial 1 twenty five local crossbreed 6–9 months old goats were bought and kept under nematode free condition but in winter season and whole trial 1 was repeated. Additionally, regulation of the TGF-β/Smad signaling pathway was evaluated only in trial 2.

### ESPs production *in vitro*

The group-1 was used to produce ESPs *in vitro* after confirmation of infection. All goats were euthanized at 28 DPI and abomasum was removed. Mixed adult worms (male and female) were collected from the abomasum, washed several times with PBS and kept in RPMI 1640 medium (100 worms/ml) contain penicillin (100 IU) and streptomycin (0.1 mg/ml; Pen strep, Gibco, Life Technologies) at 37°C under 5% CO. After 4 hours incubation, medium was changed with a new medium containing 2% glucose and incubated overnight. The next day, supernatant collection was done through centrifugation followed by filtration using a 0.2 μm pore size membrane filter. HcESPs were collected and desalted (10 mM Tris, NaCl pH7.4) by passing through a 3kDa filter (Centripep YM-3, Millipore). The protein concentration was checked by the bradford method [27].

### Separation of PBMCs

PBMCs were separated from collected blood by standard Ficoll-Paque (GE Healthcare, Munich, USA) gradient centrifugation method as described previously [28,29]. After washing twice with Ca2+/Mg2+-free PBS (pH 7.4), PBMCs were adjusted to the required density (1x10^6^ cell/ml) in culture medium (RPMI 1640) containing 10% heat-inactivated fetal bovine serum (FBS), 100 U/ml penicillin or 100 mg/ml streptomycin (GIBCO, Paisley, UK).

### Determination of goat PBMCs‑derived Th9 cells

Flow cytometry was used to determine the generation of Th9 cells *in vitro* and *in vivo*. *In vitro* experiment collected PBMCs from goats of control group were treated with different concentrations of HcESPs **(**5, 10, 20, 40, 80; μg/mL), 10 ng/ml phorbol myristate acetate (PMA) and 1 μg/mL ionomycin (Sigma) in 24 well plate containing RPMI 1640 medium at 5% CO_2_ 37°C for 48 h, while, control group was kept untreated. Subsequently, protein transport inhibitor brefeldin A Solution (BFA; 10μg/mL; BD Biosciences) was added 4–6 hours before intracellular staining [30]. Subsequently, cells were transferred to 1.5 ml tube, centrifuged at 1500 rpm for 10 minutes, washed three with PBS and stained with surface antibodies, CD2 and CD4 (BD Biosciences, USA) for 30 minutes at 4°C. After that cells were centrifuged at 500 rcf for 5 minutes at 4°C, added 500μl of fixation buffer (Beijing Solarbio Sciences & Technology Co., Ltd), and place it in dark for 20 minutes. After another washing, cells were permeabilized twice with BD Perm/Wash buffer (BD Biosciences, USA), stained with intracellular cytokine for additional 30 minutes. Consequently, cells were stained with IL-9 and IL-10 cytokine antibodies (BD Pharmingen), and flowcytometry was performed acquiring the gate at 100,000 on the FACS Canto II flow cytometer (Becton Dickinson) [30].

Furthermore, *in vivo* experiment, PBMCs were separated using the blood samples collected from P-800, P-2400, P-8000, and control group, and flow cytometry was performed at 0, 3, 5, 7, 10, 14, 18, 21, 28 DPI as described above. Additionally, flow cytometry was also performed using PBMCs separated from blood collected at 35 DPI.

### Evaluation of TGF-β/SMAD signaling

Quantitative real-time polymerase chain reaction (QRT-PCR) was used to determine the TGF-β/Smad Pathway key gene TGF-β1, TGF-BR I, TGF-BR II, Smad3, Smad4, Smad7, IRF-4 and PU.1 levels both *in vitro* and *in vivo*. PBMCs collected from blood of group -2 goats were incubated with different concentrations of HcESPs **(**5, 10, 20, 40, 80; μg/mL) in 24 well plate containing RPMI 1640 medium for 48 h at 5% CO_2_ 37°C for *in vitro* determination of TGF-β/Smad Pathway key gene level. Subsequently, the Trizol method was used to extract RNA from cell sediments using primescript RT reagent kit (Takara, CA, USA) as per manufacturer’s instruction. The cDNA-based quantifications of cytokine transcriptions were evaluated as described previously [31] using specific primer sequences (S1 Table), and β-actin was used as a reference gene (β-actin). The data were analyzed based on raw cycle threshold (Ct), obtained from the ABI Prism 7500 software (Applied Biosystems, USA) by comparative Ct (2−ΔΔ Ct) Method (26). PBMCs were separated from the blood of P-800, P-2400, P-8000 and control group’s goats at 0, 3, 5, 7, 10, 14, 18, 21 & 28 DPI and QRT-PCR was performed each day using same methods as described above. Moreover, TGF-β/Smad Pathway key genes were also evaluated using RNA extraction of PMBCs obtained from the blood of dewormed goats. The endogenous reference genes (β-actin) are present in S1 Table.

### Microscopic examination

Conventional fecal egg counting was performed with the McMaster method using fecal samples collected from P-800, P-2400, and P-8000 group at 18, 20, 22, 24, 26, 28, and 29 DPI as described previously [19]. McMaster egg counting was based on the detection of *H*. *contortus* eggs in 2g of feces dispersed in saturated NaCl (58 mL), providing a diagnostic sensitivity of 50 EPG (eggs per gram) feces.

### Determination of IL-9 cytokine

QRT-PCR was used to determine the IL-9 cytokine level both *in vitro* and *in vivo*. PBMCs collected from the blood of untreated goats were incubated with different concentrations of HcESPs **(**5, 10, 20, 40, 80; μg/mL) in 24 well plates containing RPMI 1640 medium for 48 h at 5% CO_2_ 37°C for *in vitro* determination of IL-9 level. Subsequently, the Trizol method was used to extract RNA from cell sediments using primescript RT reagent kit (Takara, CA, USA) as per manufacture’s instruction. The cDNA-based quantifications of cytokine transcriptions were evaluated as described previously [31]. The primer sequences were designed (F, GATGCGGCTGATTGTTT, R, and CTCGTGCTCACTGTGGAGT) and used for IL-9 cytokine determination. Moreover, β-actin was used as a reference gene (β-actin). The data were analyzed based on raw cycle threshold (Ct), obtained from the ABI Prism 7500 software (Applied Biosystems, USA) by comparative Ct (2−ΔΔ Ct) Method (26).

In vivo PBMCs were separated from the blood of P-800, P-2400, P-8000, and control group’s goats at 0, 3, 5, 7, 10, 14, 18, 21 & 28 DPI and QRT-PCR were performed each day using same methods as described above. Moreover, the IL-9 level was also evaluated using RNA extraction of PMBCs obtained from the blood of dewormed goats.

### Statistical analysis

Statistical analyses were performed using GraphPad Prism 7.0 (GraphPad Prism, USA). All data obtained from the above experiments were displayed as mean ± SD. One way ANOVA followed by Tukey’s *post-hoc* test was employed to compare the variances between groups and considered statistically significant at *p < 0.05, **p <0.01, ***p <0.001. Flow cytometry data were analyzed using Flow Jo (Version 10) software. To confirm the results of trial 1, both *in vitro* and *in vivo* experiments were repeated (Trial 2).

## Results

### Effects of ESPs on Th9 cells *in vitro*

**Fig 1:**
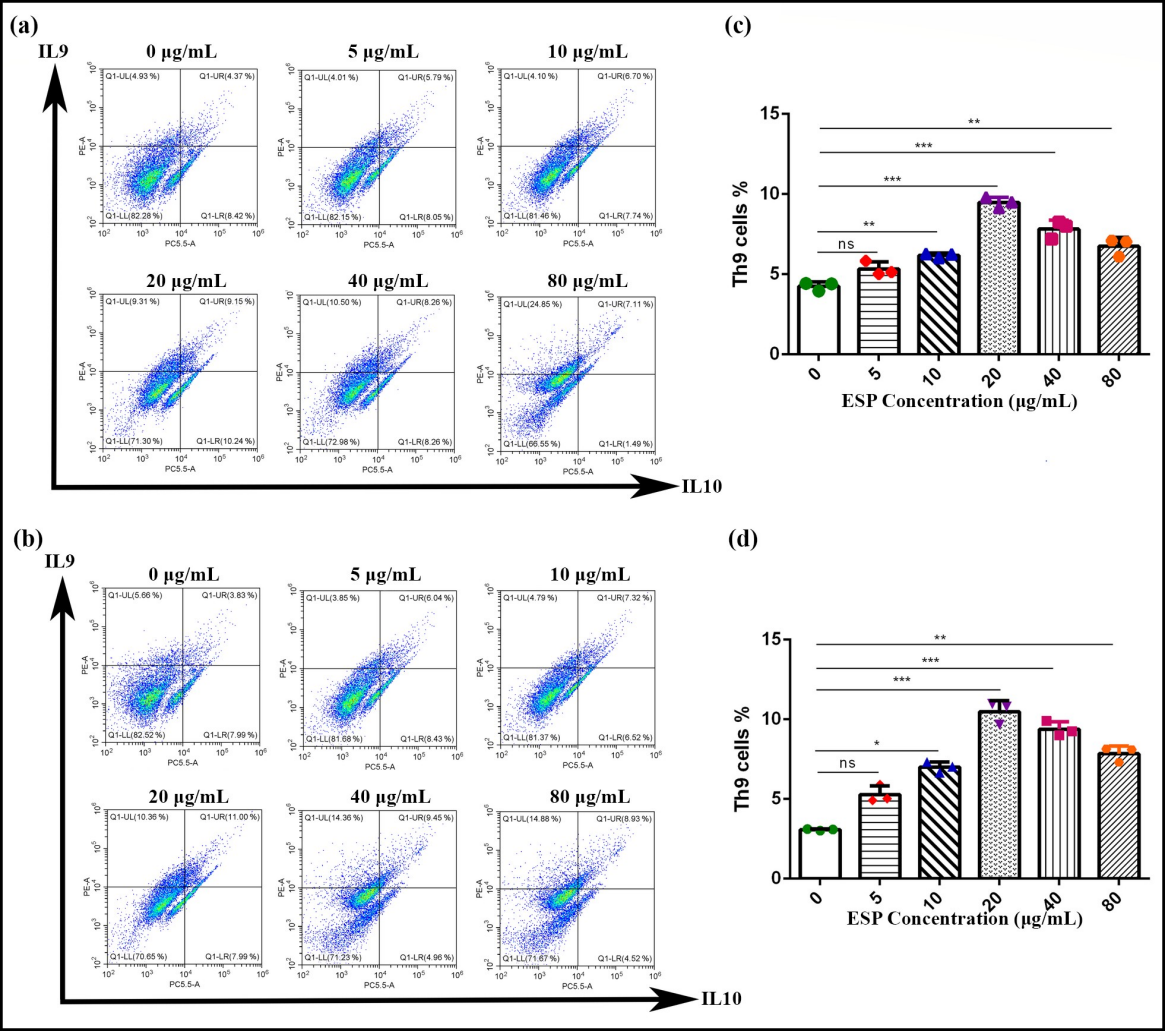
*In vitro* effects of ESPs on the population of Th9 cells by flow cytometry. **a:** Dot plot analysis (gated on CD2+CD4+ T cells) of Trial 1, PBMCs derived-T helper-9 cells treated with control (0 μg/mL) and different concentrations of ESPs (5,10, 20, 40, 80; μg/mL) using representative intracellular cytokine antibodies (IL-9 and IL-10). **b:** Dot plot analysis of trial 2; **c**:, Proportion of Th9 cells’ population in different groups of trial 1. **d**:, Proportion of Th9 cells’ population in different groups of trial 2. Data are presented of the two experiment and mean ± SD representative of triplicate experiments (*P < 0.05, (**P < 0.01, ***P < 0.001).

To evaluate the effects of ESPs on Th9 cells, different concentrations of ESPs (5, 10, 20, 40, 80; μg/mL) were incubated with goat PBMCs and flow cytometry was performed (setting the gate on CD2+CD4+T cell) by using intracellular antibodies (IL-9 and IL10) to count the Th9 cells. The flow cytometry results of two experiments revealed that 20μg/mL of ESPs showed highest percentage (9.15%) of Th9 production while, percentage production of 0, 5, 10, 40, 80; μg/mL was recorded as 4.37%, 5.79%, 6.70%, 8.26 and 7.11%, respectively (Fig 1A). Similarly in case of trial 2, 20μg/mL of ESPs showed highest percentage (11.00%) of Th9 production while, percentage production of 0, 5, 10, 40, 80; μg/mL of ESPs was recorded as 3.83%, 6.04%, 7.32%, 9.45% and 8.93%, respectively. The data showed that 10, 20, 40 and 80μg/mL of ESPs markedly increased as compared to control group (0μg/mL). Moreover, 5μg/mL of ESPs and control group were non-significant to each other (Fig 1B).

### Effects of ESPs on the regulation of TGF-β/Smad signaling *in vitro*

**Fig 2:**
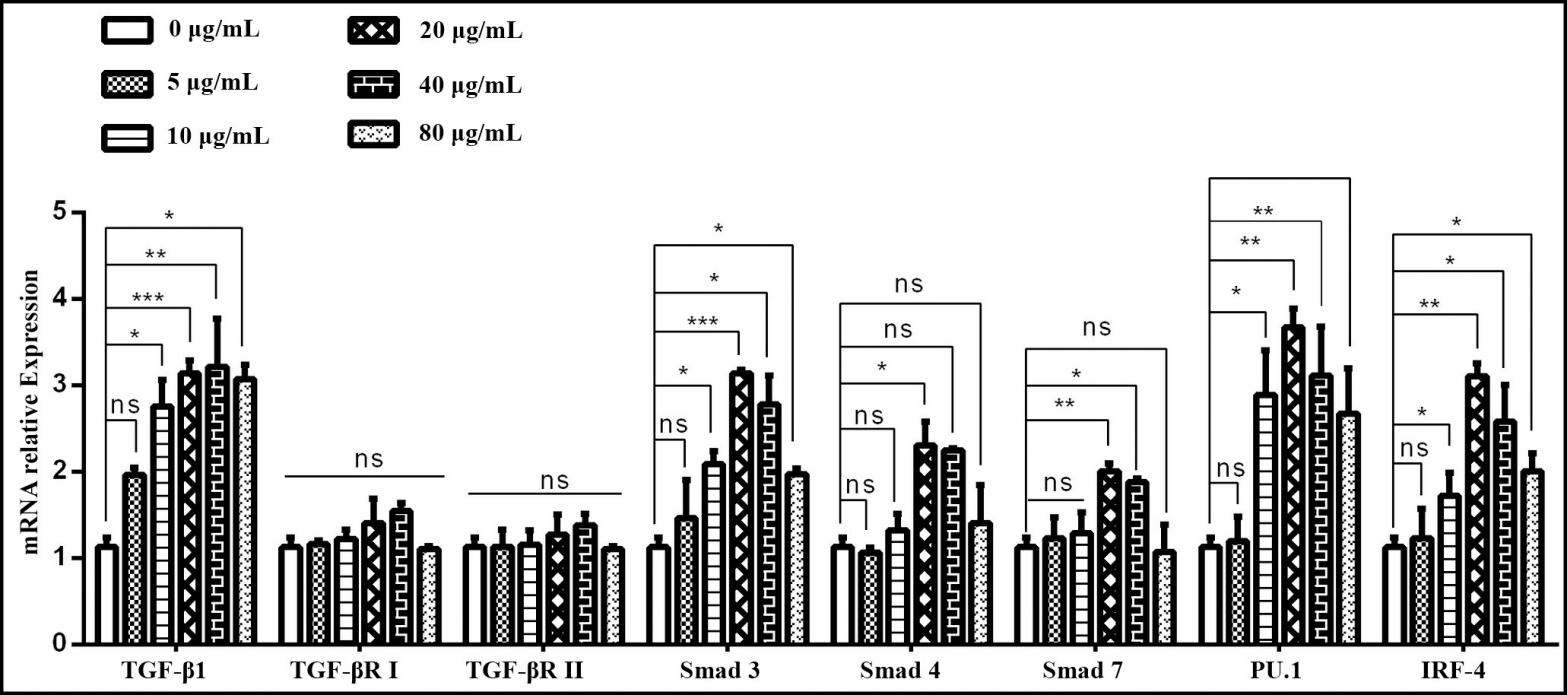
Relative expression of TGF-β/Smad signaling pathway key genes in goat PBMCs stimulated by the ESPs. Cells were incubated with the serial concentration of ESP for 48 h and the mRNAs encoding TGF-β/Smad signaling pathway key genes a reverse-transcription-polymerase chain reaction quantified TGF-β1, TGF-βRI, TGF-βRII, Smad3, Smad4, Smad7, PU.1 and IRF-4. The significant level were set at *p < 0.05, **p < 0.01, ***p < 0.001 and ns non-significant compared with the untreated group (control). Data are representative of three independent experiments.

QRT-PCR assay was performed in trial 2 only to assess the level of TGF-β/Smad signaling pathway key genes TGF-β1, TGF-βRI, TGF-βRII, Smad3, Smad4, Smad7, PU.1 and IRF-4 production by PBMCs that had been incubated with different concentrations of ESPs. The 10, 20, 40 and 80 μg/ml concentration of ESPs incubated with goat PBMCs showed significant increase in the level of TGF-β1, Smad3, and PU.1 IRF-4 transcription as compared to the control group (0μg/mL). While TGF-βRI and TGF-βRII showed no change in transcription level as compared to the control group (0μg/mL). Comparatively, 20μg/mL treatment of ESPs showed the highest increase of TGF-β/Smad signaling expression (Fig 2).

### Effects of ESPs on IL-9 cytokine *in vitro*

**Fig 3:**
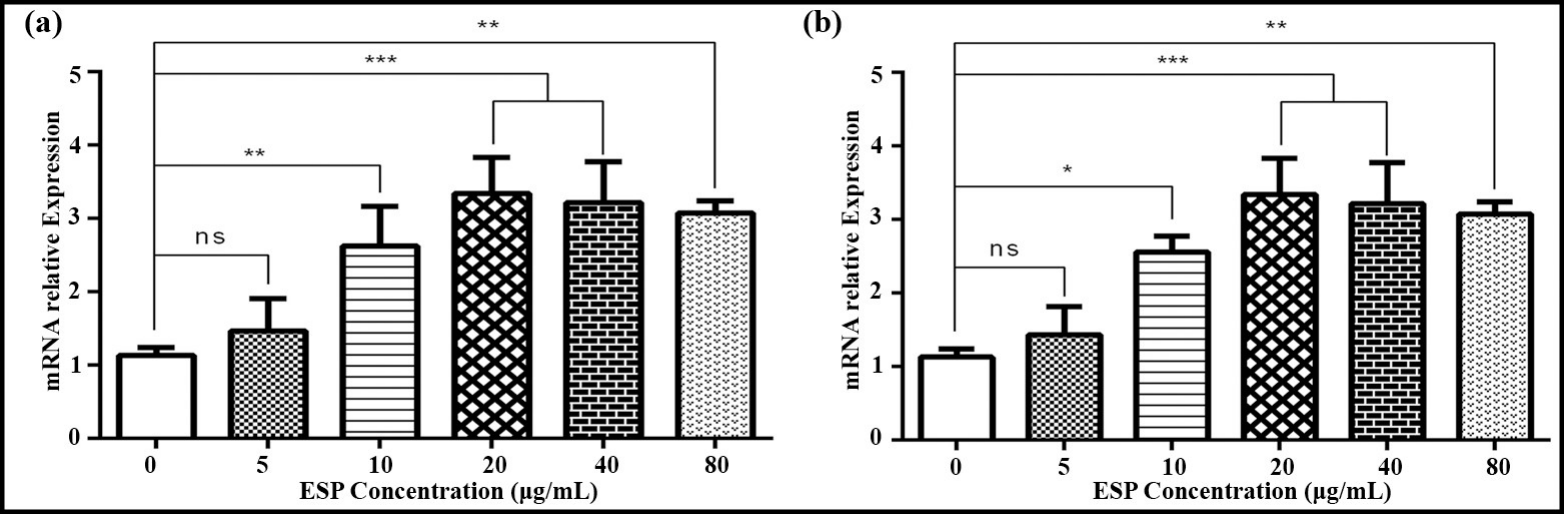
Relative expression of IL-9 cytokine in goat PBMCs stimulated by the ESPs *in vitro*. (a): IL-9 expression of trial 1; (b): IL-9 expression of trial 2. Cells were incubated with a serial concentration of ESP for 48 h, and a reverse-transcription-polymerase chain reaction quantified the mRNAs encoding interleukin-9 (IL-9). The significant level were set of two experiments at **p < 0.05, **p < 0.01, ***p < 0.001, and ns non-significant compared with the untreated group (control).Data are representative of three independent experiments.

QRT-PCR assay was performed to assess the level of cytokine production by PBMCs that had been incubated with different concentrations of ESPs. The 10, 20, 40 and 80 μg/ml concentration of ESPs incubated with goat PBMCs showed significant increase in the level of IL-9 transcription as compared to the control group (0μg/mL). Comparatively, 20μg/mL treatment of ESPs showed the highest increase of IL-9 expression and there was no significant difference between both trials (Fig 3A and 3B).

### Microscopic examination

**Fig 4:**
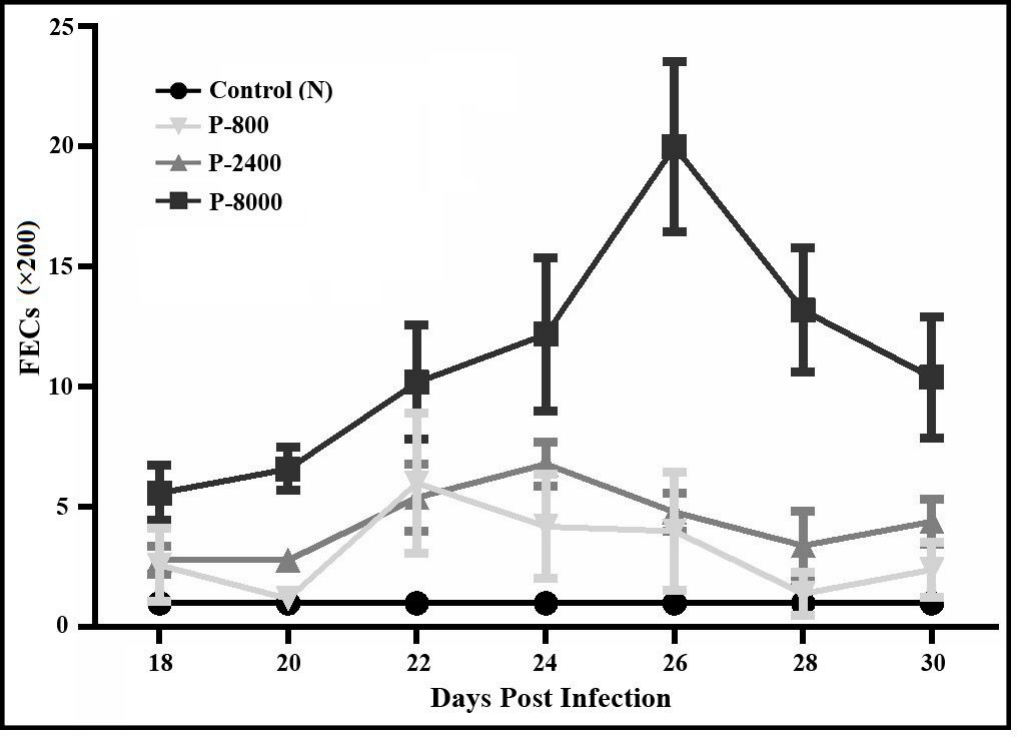
Fecal eggs count of goats at different days post-infection (DPI). Eggs were detected at 18 to 30 DPI while no eggs were detected at 0, 7 and 14 DPI.

McMaster fecal egg counts (FECs) were performed to detect the *H*. *contortus* eggs in feces collected from P-800, P-2400, P-8000 (n = 10 each), and control group at 18, 20, 22, 24, 26, 28 and 30 DPI. Mean ± SD of P-800, P-2400 and P-8000 groups were recorded as 11 ± 2.32, 4.34 ± 2.34 and 11.14 ± 5.02 respectively. P-8000 group was most prevalent of *H*. *contortus* eggs, while no eggs were detected in fecal samples of the control group (Fig 4).

### Immunomodulatory effects of *H*. *contortus* infection on Th9 cells *in vivo*

**Fig 5:**
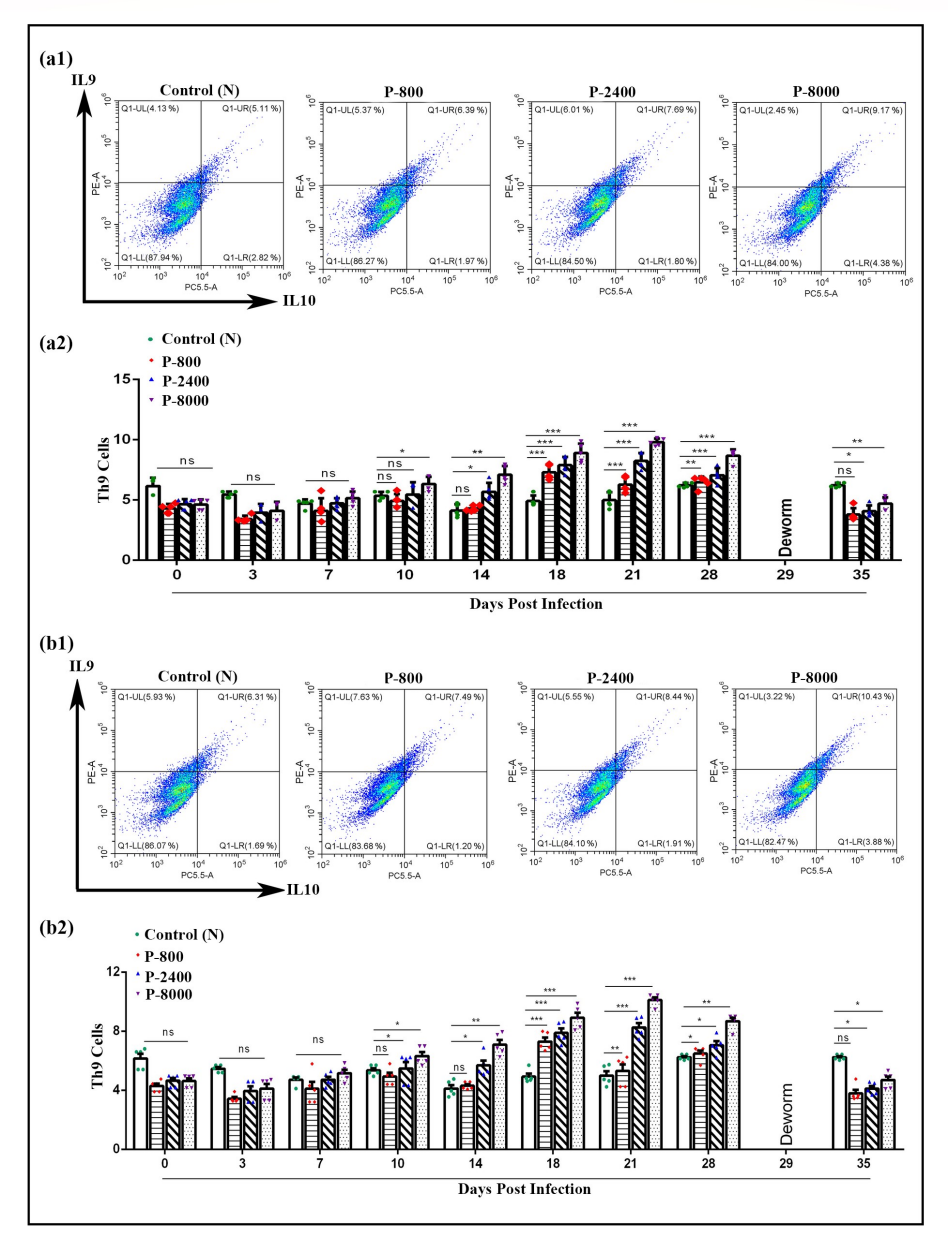
Dot plot analysis (gated on CD2+CD4+ T cells) and proportion of Th9 cells during different stages of *H*. *contortus* by flow cytometry using representative intracellular cytokine antibodies (IL-9 and IL-10). (a1): Dot plot analysis of trial 1; (a2): Th9 cells proportion of trial 1; (b1): Dot plot analysis of trial 2; (b2): Th9 cells proportion of trial 2. PBMCs derived-T helper-9 cells were collected from different groups of *H*. *contortus* infected dose rate. P-800: Goats (n = 10 each) infected with 800 *H*. *contortus* L3; P-2400: Goats infected with 2400 *H*. *contortus* L3; P-8000: Goats infected with 8000 *H*. *contortus* L3; 0–35: Days post-infection. The control group was kept untreated. Data are presented two experiment of the standard deviation (SD) and representative of triplicate experiments (*P < 0.05, **P < 0.01, ***P < 0.001).

To evaluate the effects of *H*. *contortus* infection on Th9 cells (%), PBMCs were separated using blood samples collected at 0, 3, 7, 10, 14, 18, 21, and 28 DPI from P-800, P-2400, and P-8000 and control group. Flow cytometry results revealed that no significant difference was observed between P-800, P-2400, P-8000 and control group at 0, 3 DPI. At 7 and 10 DPI, only P-8000 showed a significant increase of Th9 cells while P-800, P-2400, and control group were non-significant to each other. Moreover, at 14 DPI flowcytometry indicated the increase of Th9 cells in P-2400 (5.09%) and P-8000 group (6.65%) while no significant increase was observed between P-800 and control group. Furthermore, P-800, P-2400 and P-8000 showed a significant increase in Th9 cells as compared to the control group at 18, 21 and 28 DPI. These results suggested that Th9 cells percentage gradually increased during the *H*. *contortus* infection from 7 DPI to 28 DPI (Fig 5A1 and 5A2). Moreover, flow cytometry didn’t indicate the increase of Th9 cells after deworming (35DPI). Similar finding were observed in confirmatory trial 2 (Fig 5B1 and 5B2).

### Expression of TGF-β/Smad signaling during *H*. *contortus* infection

**Fig 6:**
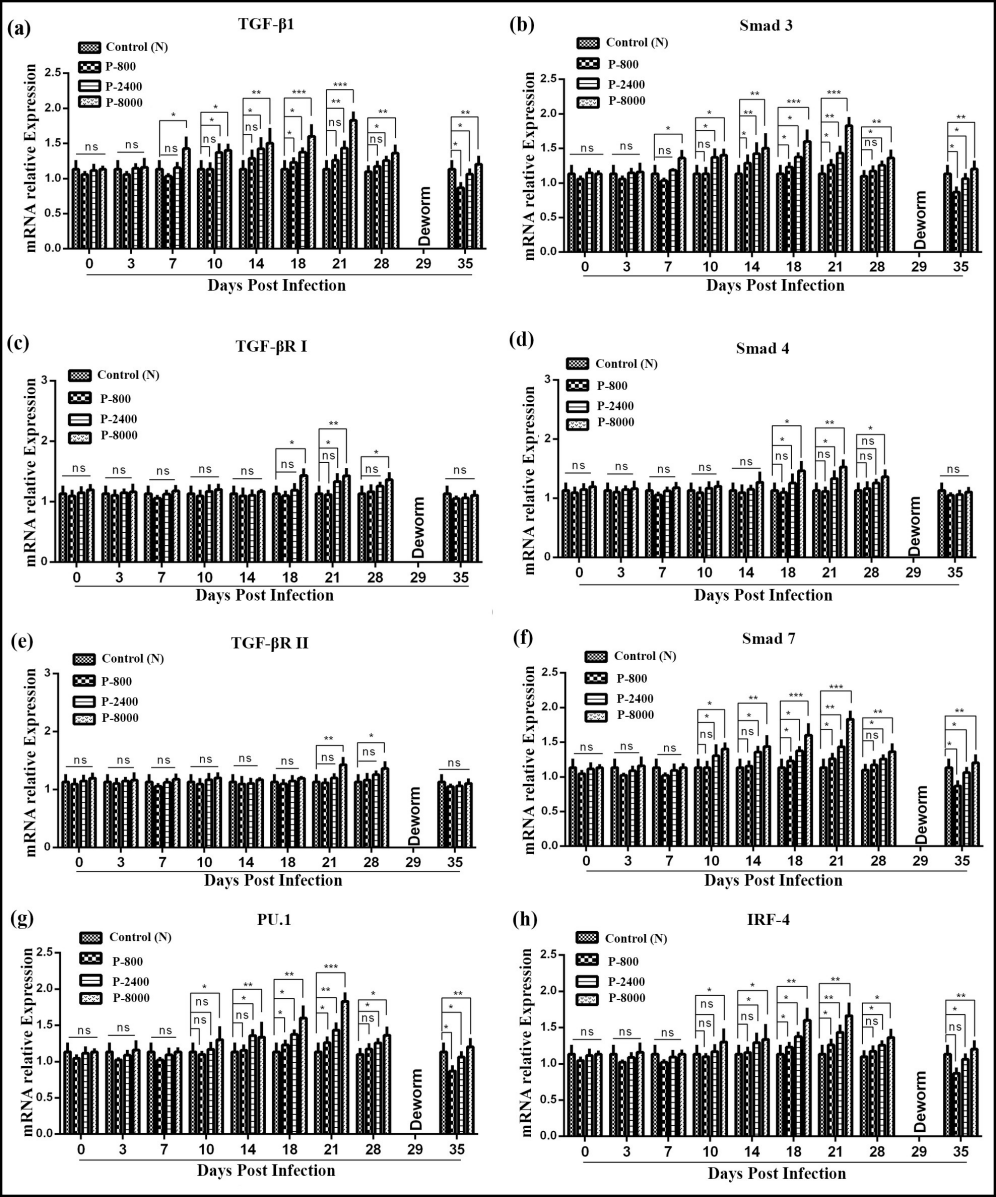
Relative expression of TGF-β/Smad signaling pathway key genes TGF-β1 (**a**), Smad3 (**b**), TGF-βRI (**c**), Smad4 (**d**), TGF-βRII (**e**), Smad7 (f), PU.1 (**g**), and IRF-4 (**h**) in goat PBMCs during different stages of *H*. *contortus* infection. P-800: Goats (n = 5 each) infected with 800 *H*. *contortus* L3; P-2400: Goats infected with 2400 *H*. *contortus* L3; P-8000 Goats infected with 8000 *H*. *contortus* L3; 0–35: Days post-infection. The control group was kept untreated. The significant level was set at *p < 0.05, **p < 0.01, ***p < 0.001, and ns non-significant compared with the untreated group (control). Data are representative of three independent experiments.

Determination of TGF-β/Smad signaling key genes (TGF-β1, TGF-βRI, TGF-βRII, Smad3, Smad4, Smad7, PU.1, and IRF-4) expression during *H*. *contortus* infection of trial 2, QRT-PCR was performed at 0, 3, 7, 10, 14, 18, 21 and 28 DPI after the collection of PBMCs from goats of P-800, P-2400, P-8000 and control groups. QRT-PCR analysis revealed that at 0 and 3 DPI, the transcription level of all key genes was non-significant (p > 0.05) in all groups. At 7, 10 and 14 DPI, only P-8000 showed a significant increase (*P* < 0.01) in TGF-β1, Smad3, Smad7, PU.1 and IRF-4 transcription level, and P-800, P-2400, and control group were non-significant to each other. Furthermore, at 18, 21 and 28 DPI, significan increase (*P* < 0.001) in the transcription level of TGF-β1, Smad3, Smad7, PU.1 and IRF-4 signaling was observed in P-800, P-2400 and P-8000 groups as compared to control group. Moreover, TGF-βRI showed significant up-regulated transcription level at 18, 21 and 28 DPI and TGF-βRII showed up-regulated transcription level at 21 and 28 DPI only in the P-8000 group as compared to control. Moreover, a significant decrease in the transcription level of TGF-β1, TGF-βRI, TGF-βII Smad3, Smad4, PU.1 and IRF-4 was observed in all groups at 35 DPI, after deworming (Fig 6).

### IL-9 expression during *H*. *contortus* infection

**Fig 7:**
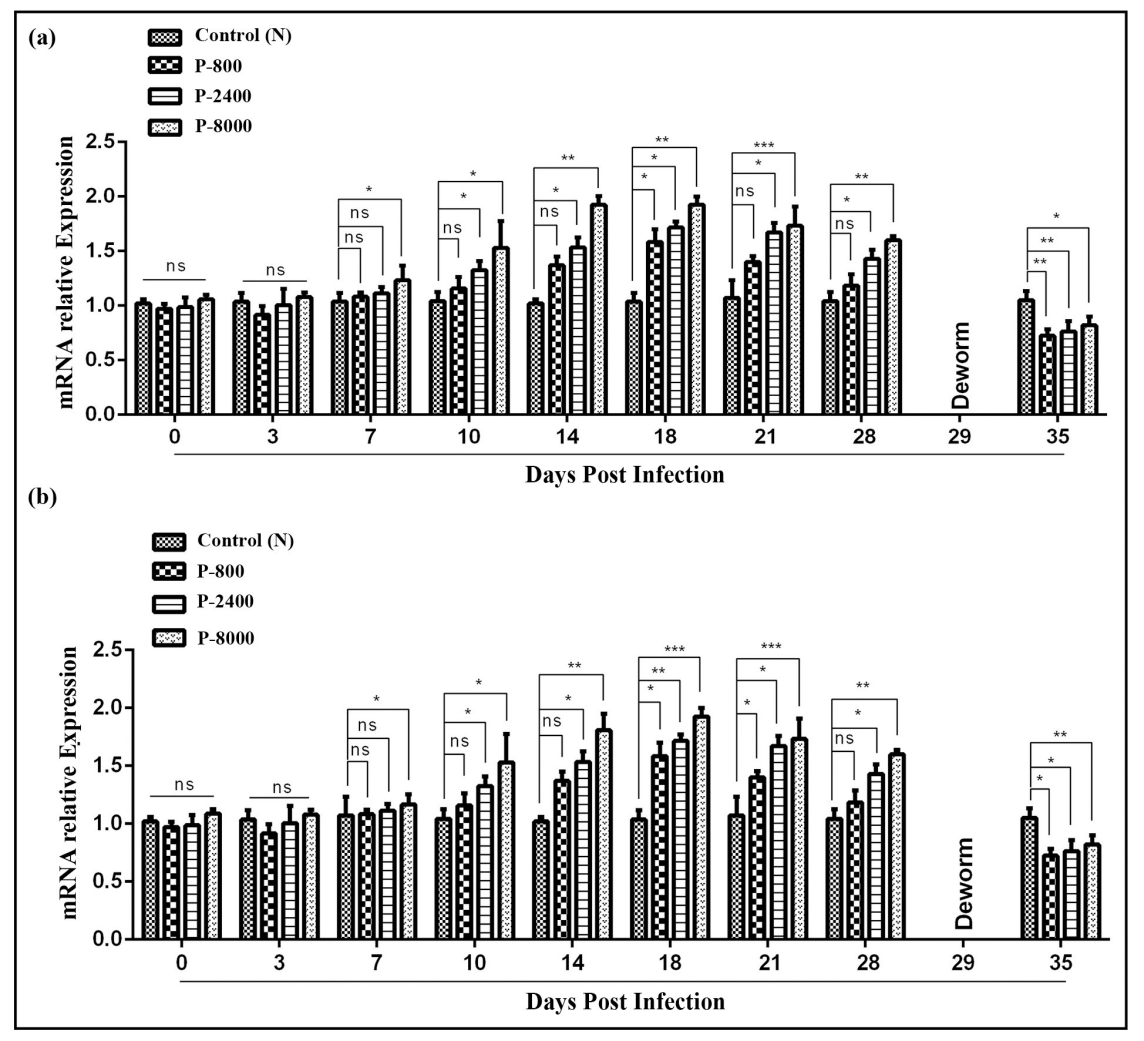
Relative expression of IL-9 cytokine in goat PBMCs during different stages of *H*. *contortus* infection. (a): IL-9 expression of trial 1; (b): IL-9 expression of trial 2. P-800: Goats infected with 800 *H*. *contortus* L3; P-2400: Goats infected with 2400 *H*. *contortus* L3; P-8000: Goats infected with 8000 *H*. *contortus* L3; 0–35: Days post-infection. The control group was kept untreated. The significant level was set at *p < 0.05, **p < 0.01, ***p < 0.001, and ns non-significant compared with the untreated group (control). Data are representative of three independent experiments.

To Determination the IL-9 expression at different stages of *H*. *contortus* infection, QRT-PCR was performed at 0, 3, 7, 10, 14, 18, 21 and 28 DPI after the collection of PBMCs from goats of P-800, P-2400, P-8000 and control group. QRT-PCR analysis revealed that at 0 and 3 DPI, IL-9 cytokine transcription level was non-significant in all groups (p > 0.05). At 7, 10, and 14 DPI, only P-8000 group showed a significant increase of IL-9 cytokine transcription level while P-800, P-2400 and control groups were non-significant to each other. Furthermore, at 18, 21 and 28 DPI significant increase in IL-9 cytokine transcription level was observed in P-800, P-2400, and P-8000 group as compared to the control group. Moreover, a significant decrease of IL-9 cytokine transcription level was observed in all groups after deworming (Fig 7A). Moreover, results of IL-9 expression at different stages of *H*. *contortus* infection in trial 2 confirm the results of trial 1 (Fig 7B).

## Discussion

CD4+ T cells differentiate into subsets of T helper cells that have been exposed to a specialized cytokine [32]. The identification of CD4+ T subsets has improved our understanding of adaptive immunity based on their cytokine secretion and immune regulatory function.[33]. The primary function of cytokines is to regulate inflammation and the immune response in disease and health [34]. The previous research of defense mechanisms against helminths infections is typically associated with a type 2 immune response characterized by the expression of the cytokines IL-3, IL-4, IL-5, IL-9, IL- 10, and IL-13 [35]. The Th9 cells are a new subtype of a subset of CD4+ T cells producing the cytokine IL-9 and play important roles in promoting effective immunity against tumor and parasites infection in mice [1]. IL-9 secreting CD4+ T cells are a predominant source of IL-9 in allergic inflammation and anti-parasite immunity [32,36]. Previously, Th9 cells have been identified as responsible for IL-9 expression *in vitro* [30]. The role of IL-9 in helminth infection was first suggested by animal studies in which IL-9 transgenic mice infected with *Trichuris muris* or *Trichinella spiralis* showed increased Th2 response and faster expulsion of the parasite from the intestine [16,37,38].Furthermore, a recent report also suggested that IL-9 plays important roles in promoting ILC2 survival and function in helminths-induced lung inflammation [39,40]. Th9 cells in human diseases were known to contribute in both protective immune responses and pathological responses leading to immune-mediated pathology [7]. Moreover, allergen-specific induction of IL-9 in PBMCs of atopic patients and in animal models has been reported [41]. *H*. *contortus* ESPs play crucial roles in parasite virulence as well as host-parasite interactions, mainly through the modulation of the host immune response [21,41]. In this study, the modulatory effects of ESPs on goat PBMCs-derived Th9 cells production and immune response *in vitro* and during different stages of *H*. *contortus* infection in goat were evaluated. Production level of Th9 cells was markedly increased in groups treated with 10, 20, 40, and 80μg/mL of ESPs while no increase was observed in the group treated with 5μg/ml as compared to control. Similarly, *in vitro* study revealed that ESPs showed significantly increased IL-9 expression level as compared to the control group. The increased IL-9 expression level was due to the up-regulation of transcription level of TGF-β/Smad pathways as a variety of immune cells and signalling molecules were involved in the chronic infection process [42–45]. The Previous research showed that TGF-β, p-Samd3, PU.1.IRF-4 and IL-9 were increased in *Echinococcus granulosus* patients with active cysts and that the TGF-β/Smads pathway regulated Th9 cells differentiation [46]. In *Schistosoma mansoni* infection, TGF-β/Smad pathways are thought to transduce signals to the cell nuclei to regulate gene expression. [47]. Similarly, previous research reported that both the mRNA and protein levels of TGF-β, TGF-βR, Smad3 and Smad4 were up-regulated in CE patients with active stages but the expression of Smad7 was decreased [48]. Respectively, current research were showed that modulatory effects of ESPs *in vitro* study, revealed that different concentrations (10, 20, 40, and 80μg/ml) of ESPs significant increase of expression level TGF- β/Smad signaling pathway key genes TGF-β, Smad3, Smad4, PU.1 and IRF-4 as compared to control group. Furthermore, the remaining key genes TGF-β RI, TGF-β RII, non-significant and also Smad7 gene expression levels were down-regulated to compared with the control group. While the 5μg/ml concentration of ESPs compared with the control group was non-significant to each other.

Previous research reported that the increased frequency of Th9 cells stimulation with two different parasite antigens (SsAg, NIE) and indicated that the Th9 cells are parasite-specific and respond to recall stimulation. Furthermore, increased co-expressing frequencies of expressing IL-4/IL-9 co-expressing were also observed and concluded that CD4+ T cell subsets also respond to *Strongyloides stercoralis* infection by enhancing IL-9 expression [30]. In another study, IL-9 produced by non-Th2 CD4+ T cell subset during early *Nippostrongylus brasiliensis* infection in mice provided sufficient host protection against worm infection [25]. Previously, the regulation of Th9 cells in *S*. *stercoralis* infection was evaluated by comparing frequencies of Th9 cells at baseline and following antigen-stimulation in infected with uninfected control individuals [30]. Respectively, in current research, the expression of IL-9 cytokine and population of Th9 cells during different stages of *H*. *contortus* infection (0, 3, 7, 10, 14, 18, 21 and 28 DPI) were gradually increased during the 7 DPI to 28 DPI and decreased population of Th9 cells was observed after deworming (35DPI). Moreover, the expression of IL-9 confirmed the increased production of Th9 cells by gradually from 7 DPI to 28 DPI. The previous study reported that *Schistosoma mansoni* infection could induce IL-9 in mice [49]. Moreover, a high percentage of IL-9-secreted CD4+ Th9 cells was kinetically observed in *Schistosoma japonicum-* infected mouse liver by intracellular cytokine staining. The percentage of Th9 cells increased on week 4 after infection and reached the maximum on week 5 after infection [50]. Furthermore, previous research reported an increased expression of TGF-βR, Smad3 mRNA and especially Smad4 which is a central mediator in TGF-β superfamily signaling [20]. Few differences between RNA expression and the amount of protein regarding TGF-b R and Smad may explain by posttranscriptional events and deserve further studies since parasite components could cause such events.[46] Another the study showed that the expression of Smad4 was higher in areas surrounding lesions than in distant liver in the patients with AE. Smad7, which is induced by TGF-β itself, is responsible for the fine-tuning of TGF-b signals [36]. It prevents the phosphorylation of Smad proteins, associates with ubiquitin ligases involved in TGF-b R-degradation, and acts as a transcriptional repressor inhibiting Smad-dependent promoter activation [37]. Furthermore, the study also excluded the possibility that endogenously produced TGF-β or endogenous TGF-β superfamily member signalling in naive CD4+ T cells contribute to IL-9 production in Th9 IL-4+IL-1β cells. Respectively, in current research, the expression of TGF-β/Smad pathway key genes was regulated during different stages of *H*. *contortus* infection (0, 3, 7, 10, 14, 18, 21, and 28 DPI).

IL-9 is an important regulator that drives mucosal Type 2 immunity *in vivo*[33]. Moreover, by using two different methodologies, the previous report verified the important association of enhanced Th9 responses and its reversibility following treatment during *S*. *stercoralis* infections. While we have demonstrated statistically significant changes in Th9 immune response. Moreover, further study is needed to evaluate the actual mechanism of Th9 immunodynamics Another study indicated that the effect of IL-9 to induce inflammation in *S*. *Japonicum-*infected mouse liver is limited and related to the powerful function of IL-9 in recruiting inflammatory cells [51]. Furthermore, the failure to mount an enhanced immune response against *T*. *spiralis* infection in IL-9- deficient mice was reported [52]. To the best of our knowledge, this is first study to report Th9 immune response during *H*. *contortus* infection in goat. This study has demonstrated that Th9 immune response was induced *in vitro* and *in vivo* during *H*. *contortus* infection in goat by up-regulation of TGF-β/Smad signaling key genes.

## Supporting information

S1 Table Primer sequences for quantitative real-time PCR. (DOCX)
