# Supplementary material for: Immunomodulatory dynamics of excretory and secretory products on Th9 immune response during Haemonchus contortus infection in goat
Source: PLoS Negl Trop Dis. 2020 Apr 3;14(4):e0008218. doi: 10.1371/journal.pntd.0008218 (PMC7159227; doi:10.1371/journal.pntd.0008218)
Supplement: S1 Table — (DOCX) [file pntd.0008218.s001.docx]

| Name of Genes | Forword5 3 | Reverse 5 3 | Amplification  Size (bp) | Amplification  Efficiency (%) |
| --- | --- | --- | --- | --- |
| Beta-Actin | CACCACACCT TCT ACAAC | TCTGGGTCATCTTC  TCAC | 106 | 95.41 |
| IL-9 | GATGCGGCTGAT  TGTTT | CTCGTGCTCACTGT GGAGT | 103 | 98.65 |
| TGF-β1 | CACGAATGGCT  CCACATAA | GATTTGCAGGTATT  GATGGCAC | 123 | 93.24 |
| TGF-βRI | ACCAGGACCACT  GCAATAAA | GTATCTCACTCATG  CTGATGGT | 142 | 96.89 |
| TGF-βRII | CAGAACACGTCT  GAGCAGTT | CATCTTCTCGGACA  TCAACCTC | 105 | 98.87 |
| Smad3 | CAGATGAACCAC  AGCATGGA | TTCTGGTGCTCCATC  TCCTA | 128 | 94.91 |
| Smad4 | GGCAGCCATAGTG  AAGGATT | CATAACAGCACTAC  CACCTGG | 114 | 97.67 |
| Smad7 | CCCTCCTCCTTACT  CCAGATAC | GGGAACGAATTATCT  GGCCC | 107 | 95.56 |
| IRF-4 | GACATCTCAGACC  CGTACAAAG | ATGACAACGCCTTAC  CCTTC | 131 | 98.13 |
| PU.1 | GGAGCCCGGCTGG  ATGTTAC | CACCAGGTCTTCTGAT  GGCTGA | 118 | 93.25 |

**Table S1.Primer sequences for real-time PCR**
